# Comprehensive evaluation of the water-fertilizer coupling effects on pumpkin under different irrigation volumes

**DOI:** 10.3389/fpls.2024.1386109

**Published:** 2024-04-19

**Authors:** Tao Zhong, Jinxia Zhang, Liangliang Du, Lin Ding, Rui Zhang, Xingrong Liu, Fangfang Ren, Meng Yin, Runheng Yang, Pengliang Tian, Kaiyuan Gan, Tian Yong, Qirun Li, Fuqiang Li, Xuan Li

**Affiliations:** ^1^ College of Water Conservancy and Hydropower Engineering, Gansu Agricultural University, Lanzhou, Gansu, China; ^2^ Gansu Research Institute for Water Conservancy, Lanzhou, Gansu, China; ^3^ Geological Hazards Prevention Institute, Gansu Academy of Sciences, Lanzhou, Gansu, China

**Keywords:** irrigation volume and organic fertilizer amount, water-fertilizer coupling, agricultural water management, water-fertilizer use efficiency, pumpkin yield, comprehensive evaluation

## Abstract

Compared to conventional irrigation and fertilization, the Water-fertilizer coupling can significantly enhance the efficiency of water and fertilizer utilization, thereby promoting crop growth and increasing yield. Targeting the challenges of poor crop growth, low yield, and inefficient water and fertilizer utilization in the arid region of northwest China under conventional irrigation and fertilization practices. Therefore, a two-year on-farm experiment in 2022 and 2023 was conducted to study the effects of water-fertilizer coupling regulation on pumpkin growth, yield, water consumption (ET), and water and fertilizer use efficiency. Simultaneously the comprehensive evaluation of multiple objectives was carried out using principal component analysis (PCA) methods, so as to propose an suitable water-fertilizer coupling regulation scheme for the region. The experiment was set up as a two-factor trial using water-fertilizer integration technology under three irrigation volume (W1 = 37.5 mm, W2 = 45.5 mm, W3 = 52.5mm) and three organic fertilizer application amounts (F1 = 3900-300 kg ha^-1^, F2 = 4800-450 kg·ha^-1^, F3 = 5700-600 kg·ha^-1^), with the traditional irrigation and fertilization scheme from local farmers as control treatments (CK). The results indicated that irrigation volume and organic fertilizer application significantly affected pumpkin growth, yield, and water and fertilizer use efficiency (P<0.05). Pumpkin yield increased with increasing irrigation volume. Increasing organic fertilizer levels within a certain range benefited pumpkin plant growth, dry matter accumulation, and yield, however, excessive application beyond a certain level had inhibited effects on those. The increased fertilizer application under the same irrigation volume enhanced the efficiency of water and fertilizer utilization. However excessive irrigation only resulted in inefficient water consumption, reducing the water and fertilizer use efficiency. The Comprehensive evaluation by PCA revealed that the F2W3 treatment outperformed all the others, effectively addressing the triple objectives of increasing production, improving efficiency, and promoting green production. Therefore, F2W3 (Irrigation volume: 52.5 mm; Fertilizer application amounts: 4800-450 kg/ha^-1^) as a water and fertilizer management scheme for efficient pumpkin production in the arid region of northwest China.

## 1 Introduction

Pumpkin, is an annual herb with a long cultivation history and wide distribution. China is the largest producer and consumer in the world. According to the latest FAO statistics, global pumpkin production in 2021 reached 2.38 million tons, with China accounting for 31.2% at 0.74 million tons (FAO, 2021). Due to its rich raw materials and health benefits, pumpkins offer great economic advantages (Rico et al., 2020; Zeng et al., 2023).

In the traditional planting process, over-irrigation and fertilization are common, leading to the waste of water and fertilizer resources and a decline in crop yield (Wang et al., 2021), thus restricting the sustainable development of the pumpkin planting industry. Therefore, it is great significant to carry out the research on reasonable water-fertilizer coupling regulation of pumpkin for its green and efficient production.

Water-fertilizer coupling effect is defined as the impact of the interaction relationship between nitrogen (N), phosphorus (P), potassium (K) and other elements contained in water and fertilizers (Cheng et al., 2023; Liu et al., 2023), which on crop growth and development, yield formation and its water-fertilizer use efficiency, in agricultural ecosystems (Liu et al., 2019). That can be classified into positive effect of mutual reinforcing (i.e., synergistic effect), negative effect of mutual offsetting (i.e., antagonistic effect), and the effect of no avail, the sum of the effects of each system (i.e., superimposed effect) (Yu et al., 2019). The use of water-fertilizer coupling technology practice in production can achieve the best combination of water and fertilizer (Koc and Nzokou, 2023), which is a powerful measure to promote high-quality sustainable development of farmland (Zhang et al., 2017a). A scientific and reasonable water and fertilizer management system can not only improve crop water consumption and water and fertilizer use efficiency (Cai et al., 2023; Wu et al., 2023), but also holds significant academic significance and practical value for improving crop yield (Dou et al., 2022; Huang et al., 2023).

In summary, although there have been many studies on water-fertilizer coupling effect at present, but most of them are focused on wheat, fruit trees, corn, and other crops (Chen et al., 2016; Li et al., 2023). Research on the mechanism of water-fertilizer coupling effect on the physiological growth, yield and water-fertilizer use efficiency of pumpkin is still vacant or not yet fully explored, which is very important for the research on green and efficient production of pumpkin. In this paper, to adequately fill the knowledge gap in the study, we focus on the potential of water-fertilizer coupling regulation to replace the traditional irrigation and fertilization scheme for yield increase and efficiency in pumpkin, will be explored in depth. Specifically, by a two-year field trial, this research would be conducted to study the regulation mechanism of different water-fertilizer coupling schemes on the physiological growth, yield and water-fertilizer use efficiency of pumpkin in the northwest arid region. Moreover, it will be also combined with principal component analysis, correlation analysis and cluster analysis to seek the optimal water-fertilizer scheme, so that the goals of increased yield and efficiency, green production, on pumpkin in the region, can be achieved.

## 2 Materials and methods

### 2.1 Overview of the experimental area

**Figure 1:**
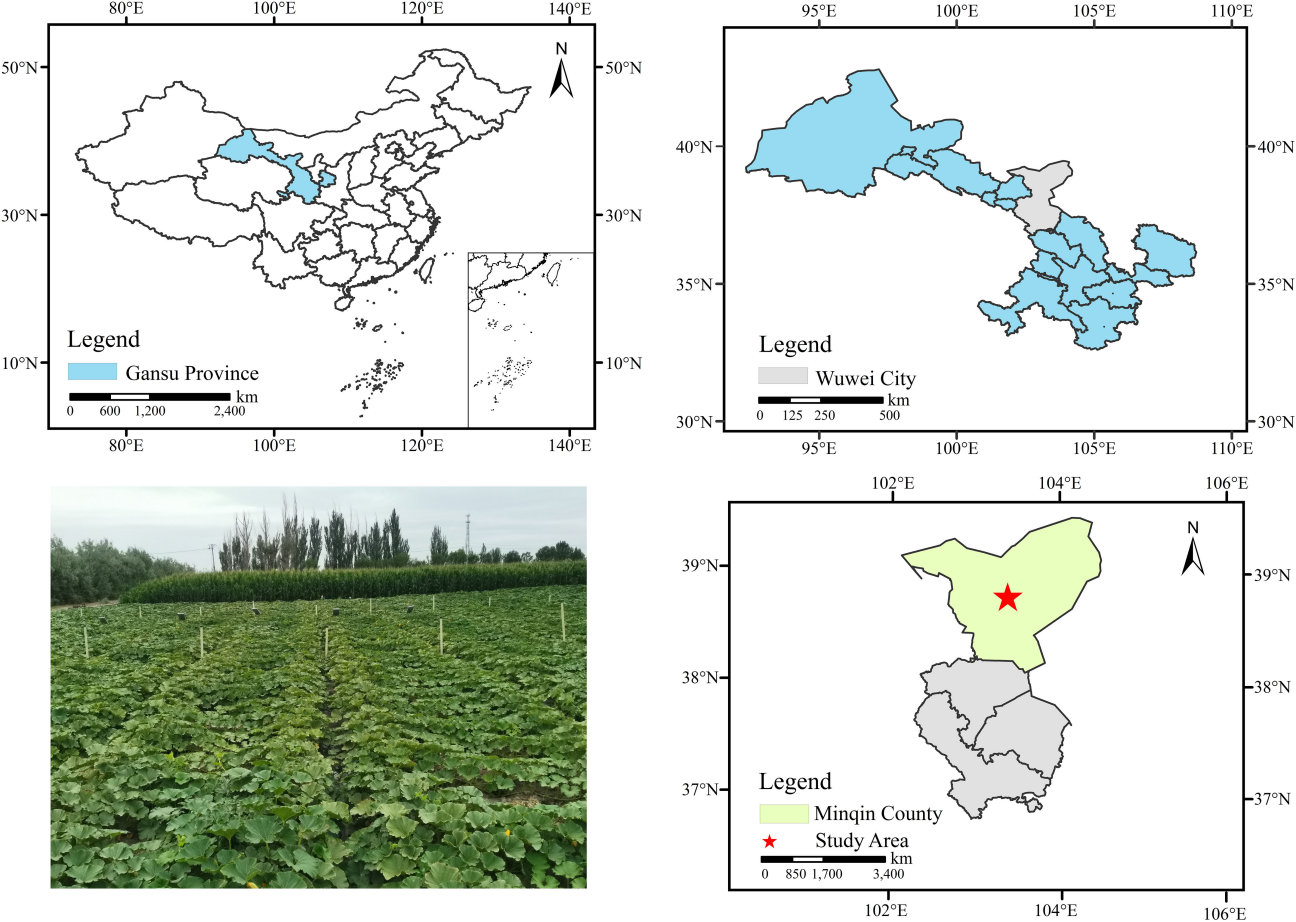
Geographical location of the test area.

The experiment was conducted in 2022 and 2023 at Minqin Irrigation Experimental Station of Gansu Research Institute for Water Resources, China (103°08´ E, 38°37´ N). The experimental geographic location is shown in **Figure 1**. The station is in Dongda Village, Datan Township, about 13.5 km north of Minqin County, Gansu Province, which is situated at the junction of the oasis and the Tengger Desert. With an average elevation of 1400m, it belongs to a typical continental desert climate. The soil used for the test is clay loam at a depth of 0~60cm, and gradually changes to sandy loam below 60cm, with an average dry bulk weight, specific gravity, porosity, field water holding capacity, and permanent wilting point of 1.54 g·cm^-3^, 2.61 g·cm^-3^, 42.80%, 35.42%, and 7.65% in the 0-100cm soil, respectively. The available water content of 0-60cm soil was 144.45mm.

### 2.2 Experimental design

In this experiment, based on previous research (Liu et al., 2014; Li et al., 2022), and considering the climate, soil characteristics, local irrigation and fertilization scheme for pumpkin under conventional furrow irrigation in the experimental area, two control factors were selected: irrigation volume and organic fertilizer amounts. The experiment followed a two-factor randomized block design with three levels for each factor, namely low, medium and high, increasing sequentially in quantitative order. The three levels irrigation of volumes were W1 (37.5 mm), W2 (45.0 mm) and W3 (52.5 mm). For the three levels of organic fertilizer amounts, solid bottom fertilizer-liquid fertilizer application amounts were F1 (3900-300 kg ha^-1^), F2 (4800-450 kg ha^-1^), and F3 (5700-600 kg ha^-1^), respectively, with the liquid trailing fertilizer application twice during the growth period, of which effective components are: N+P_2_O_5_+K_2_O ≥ 18% effective bacteria (Bacillus subtilis + Bacillus licheniformis) ≥ 50 million/ml, and amino acids ≥ 3% (Lanzhou Xindali Water Fertilizer Integrated Service Co., Ltd.). Additionally, the conventional irrigation and chemical fertilization scheme from local farmers were chosen as control treatments (CK), including an irrigation volume of 52.5 mm along with bottom fertilizer of 300 kg ha^-1^ diammonium phosphate (DAP) (0-46-0) and 450 kg ha^-1^ urea (with N ≥46.3% or 46-0-0, Lanzhou Zhongshi fertilizer Co., Ltd), also trailing fertilizer of 300 kg ha^-1^ urea twice. Therefore, there are a total of 10 treatments replicated three times, 30 plots of 87.5 m^2^ (35 m × 2.5 m). Each treatment underwent harrowing, leveling, and furrowing before sowing, and being irrigated once before sowing, with an irrigation volume of 45.0 mm. It was irrigated thrice during the growth period following the same irrigation frequency and irrigation time for all treatments during the entire growth period. The specific experimental design program is shown in **Table 1**.

**Table 1:** Experimental design scheme.

Treatment	Irrigation volumes (mm)		Times of Irrigation in the growth period	Irrigation quota (mm)	Base fertilizer		Follow up fertilizer	
	Before sowing	the growth period			Type	Quota (kg ha^-1^)	Frequency	Amounts (kg ha^-1^)
F1W1	45	37.5	3	157.5	Solid organic fertilizer	3900	Liquid Organic Fertilizer; 2 times	300
F1W2	45	45.0	3	180.0		3900		300
F1W3	45	52.5	3	202.5		3900		300
F2W1	45	37.5	3	157.5		4800		450
F2W2	45	45.0	3	180.0		4800		450
F2W3	45	52.5	3	202.5		4800		450
F3W1	45	37.5	3	157.5		5700		600
F3W2	45	45.0	3	180.0		5700		600
F3W3	45	52.5	3	202.5		5700		600
CK	45	52.5	3	202.5	Diammonium phosphate	300	Urea, 2 times	300
					Urea	450		

The test variety is “Sweet Pumpkin”, which is a conventional cultivar by local farmers. Pumpkin Seeds for the test were sown on April 28, 2022, and April 29, 2023, respectively. Additionally, pumpkin was harvested, at once tested for yield measurement on August 15, 2022, and August 20, 2023, respectively. The seeds were sown at a spacing of 200 cm between large rows, 50cm between small rows, with a plant spacing of 30cm, 2 rows plant in one furrow and one film, and 1~2 plants per hole. The test site was deeply plowed, and winter irrigated during the leisure period, with an irrigation volume of 120.0 mm.

### 2.3 Measurement indicators and methods

#### 2.3.1 Growth dynamics indicators

Vine length: At the end of each growth period of pumpkin, 10 plants were selected from each treatment to measure the vine length, with a steel tape measure of 1 mm accuracy, and then averaged.

Stem thickness: At the end of each growth period of pumpkin, 10 plants were selected from each treatment to measure stem thickness, with a vernier caliper of 0.02 mm accuracy, and the average value did take.

Leaf area: 5 plants were sampled from pumpkin seedling stage to maturity in each experimental plot fixation and determined by portable leaf area meter (ECA-YM02 type, YIKANGNONG Co., Ltd, China).

#### 2.3.2 Dry rate

During each growth period of pumpkin, 5 plants were randomly taken from each treatment to measure rhizome fresh weight, leaf fresh weight and total fresh weight, respectively. After marking in sequence, the samples were put into sample bags and put into the oven at 105°C for 30 minutes, then dried at 55°C until a constant weight. Obtaining the dry weights with rhizome and leaf, and total dry weight, respectively, followed. Finally, the drying rate was calculated.


Dr=DW/FW

Where *Dr* is the drying rate; *DW* is the drying weight, g; *FW* is the fresh weight, g.

#### 2.3.3 Yield

During the pumpkin harvest, each plot was harvested individually and measured for yield and yield components. The yield of the entire planting was then calculated based on the yield of each plot.

#### 2.3.4 Water-fertilizer use efficiency

Water and fertilizer use efficiency is represented by water use efficiency (WUE), irrigation water use efficiency (IWUE) and partial factor productivity (PFP). The formulas for WUE, IWUE, and PFP are as follows, respectively:


WUE=Y/W

IWUE=Y/I

PFP=Y/F

Where *Y* is the economic yield, kg ha^-1^; *W* is the total water consumption during planting, m^3^ ha^-1^; *I* is the total irrigation water amount during planting, m^3^ ha^-1^; *F* is the total fertilizer application during planting, kg ha^-1^; Other symbols, as above.

#### 2.3.5 Weather data

The weather data were measured by the meteorological and ecological environment monitoring system (QX100, SCIENTO Technology Co. LTD, China) at the test site, with 24-hour observation in a day and automatic data recording by data collectors.

#### 2.3.6 Soil moisture monitoring

Soil moisture content was monitored at each treatment by an intelligent wireless moisture monitoring instrument (ET100, Oriental wisdom Sense Technology Co. LTD, China), which automatically collected soil moisture data every 10cm layer from 0-100cm.

The formula for soil water storage was as follows:


ΔSWS=10×V×h

Where ΔSWS is the water storage capacity, mm; *V* is the volumetric water content, cm^3^·cm^-3^; and *h* is the soil depth, cm.

Evapotranspiration (ET): In this experiment, ET was determined using the farm water balance equation, which was calculated as follows:


ET=P+I−ΔSWS

Where *ET* is the amount of transpiration evaporation, that is the total water consumption, mm; *P* is the amount of precipitation, mm; *I* is the amount of irrigation water, mm; and Δ*SWS* is the change in soil water storage between maturity and seedling period, mm. In this experiment, the terrain of the test plot is flat, the average depth of groundwater is 20 m, and the runoff and deep leakage are ignored.

### 2.4 Principal component analysis

In this experiment, we conducted principal component analysis by SPSS software (IBM US). The key principal components were selected according to the principle of principal components greater than 1 and the principal component scores were calculated through the comprehensive analysis of the growth dynamic indexes, yield and water and fertilizer use efficiency of pumpkin. Then, the contribution of each principal component was used as weights to derive a principal component composite model. Finally, the ranking of the scores between the different treatments was derived.

### 2.5 Data analysis

The software SPSS statistics 27 (IBM US) was used for mathematical and statistical analysis, comprehensive evaluation calculation, and cluster analysis. Origin 2022 (Origin Lab US) was used for drawing.

## 3 Results and analysis

### 3.1 Effect of water-fertilizer coupling regulation on pumpkin growth dynamics

#### 3.1.1 Vine length

The data in **Table 2** demonstrates the consistent impact of water-fertilizer coupling on pumpkin vine length in 2022 and 2023. Significantly different results were observed among treatments during the same growth period (P<0.01). Both irrigation and fertilization significantly affected pumpkin vine length throughout the entire growth period (P<0.01).

**Table 2:** Effect of coupled water and fertilizer regulation on Vine length in pumpkin.

Year	Treatment	Vine length (cm)			
		seedling stage	Vine stage	Flowering stage	Maturity stage
2022	F1W1	71.80 ± 3.27d	149.52 ± 10.55d	214.80 ± 7.60f	339.00 ± 4.32g
	F1W2	78.00 ± 5.15cd	160.32 ± 3.24c	236.10 ± 8.26e	381.00 ± 5.35f
	F1W3	87.80 ± 4.60cd	173.52 ± 3.35ab	262.80 ± 6.99d	420.87 ± 3.37e
	F2W1	101.20 ± 1.92bc	166.80 ± 7.15bc	270.30 ± 2.46d	374.67 ± 5.73f
	F2W2	108.40 ± 3.21ab	176.40 ± 3.5ab	306.90 ± 5.96c	412.00 ± 6.68e
	F2W3	99.80 ± 5.85a	180.72 ± 5.41a	328.20 ± 6.99ab	445.83 ± 2.19c
	F3W1	99.60 ± 3.36ab	170.40 ± 4.07abc	305.40 ± 6.84c	433.67 ± 9.88d
	F3W2	113.40 ± 5.08ab	173.28 ± 3.76ab	317.40 ± 11.98bc	471.33 ± 4.92b
	F3W3	113.60 ± 5.90ab	177.12 ± 4.84ab	339.90 ± 8.32a	508.75 ± 6.18a
	CK	110.00 ± 3.54a	161.04 ± 2.15c	214.80 ± 10.31f	385.50 ± 7.59f
2023	F1W1	60.43 ± 0.36f	129.67 ± 0.82e	209.00 ± 4.30f	337.33 ± 20.50f
	F1W2	70.22 ± 0.51e	133.33 ± 4.6de	223.67 ± 10.50 e	357.33 ± 30.44def
	F1W3	68.17 ± 0.54e	142.53 ± 2.01c	243.67 ± 1.08bcd	376.33 ± 10.21cde
	F2W1	73.17 ± 0.20d	135.00 ± 0.71d	224.33 ± 2.27e	348.67 ± 20.43ef
	F2W2	84.87 ± 0.73b	147.13 ± 1.24c	242.60 ± 3.29bcd	365.17 ± 12.69cdef
	F2W3	90.00 ± 2.12a	156.13 ± 3.22b	255.00 ± 3.67b	384.67 ± 12.66bcd
	F3W1	76.20 ± 1.36c	143.13 ± 5.28c	233.67 ± 2.86de	391.33 ± 16.58bc
	F3W2	86.00 ± 2.45b	155.67 ± 5.96b	246.67 ± 4.14 bc	413.33 ± 17.25b
	F3W3	91.67 ± 0.82a	167.67 ± 1.08a	270.00 ± 4.85a	466.33 ± 20.14a
	CK	72.23 ± 3.27d	135.33 ± 4.10d	240.73 ± 12.33cd	362.00 ± 18.09cdef
Average	F1W1	66.12 ± 1.60g	139.59 ± 4.99f	211.9 ± 4.52g	338.17 ± 12.2f
	F1W2	74.10 ± 2.57f	146.83 ± 3.55e	229.88 ± 5.22f	369.17 ± 15.12e
	F1W3	77.98 ± 2.05f	158.03 ± 2.09d	253.23 ± 3.86e	398.60 ± 6.12d
	F2W1	87.18 ± 0.91e	150.90 ± 3.70e	247.32 ± 2.26e	361.67 ± 12.20e
	F2W2	96.63 ± 1.39bc	161.77 ± 1.64cd	274.75 ± 4.6cd	388.58 ± 8.43d
	F2W3	94.90 ± 3.11cd	168.43 ± 3.37ab	291.60 ± 4.75b	415.25 ± 5.72c
	F3W1	87.90 ± 1.76e	156.77 ± 3.56d	269.53 ± 4.09d	412.50 ± 13.08c
	F3W2	99.70 ± 3.27ab	164.47 ± 3.85bc	282.03 ± 6.37bc	442.33 ± 11.09b
	F3W3	102.63 ± 3.11a	172.39 ± 2.58a	304.95 ± 4.30a	487.54 ± 10.48a
	CK	91.12 ± 1.25de	148.19 ± 2.58e	227.77 ± 8.73f	373.75 ± 9.31e

Lowercase letters in the table indicate differences between different treatments in one growth stage (p<0.05).

The vine stage was crucial for the growth of vines in this experiment. The vines grew slowly during the seedling stage, rapidly during flowering stages, and ultimately reaching its maximum at maturity. The overall growth of the vine exhibited a positive correlation with irrigation and fertilization factors. In 2022 and 2023, under the same level of irrigation volume during of pumpkin, the vine length at the maturity stage was as follows: F3>F2>F1 under fertilization influence, W3>W2>W1 under irrigation influence factors (data used the two -year average value, the same below). The vine length under F3W3 treatment reached the maximum, significantly higher than other treatments. It increased by 44.18%, and 27.50% compared to the F1W1 and CK, respectively. Compared to that at seedling stage, vine growth stage, and blooming stage, The length of F3W3 treated vines increased by 375.70%, 182.80%, and 59.60% at the maturity stage of pumpkin, compared to the first three growth stages, respectively. That of F1W1 treatment increased by 412.50%, 141.30%, and 54.50%, respectively, of CK treatment increased by 200.50%, 152.40%, and 64.20%, respectively.

It could be seen that, the organic fertilizer application increased the growth rate of pumpkin vine length compared to CK, increasing both fertilization amount and irrigation volume promoted the pumpkin vine growth.

#### 3.1.2 Stem thickness

**Table 3** shows the effect on pumpkin stem thickness, and the data trend remained consistent in 2022 and 2023. The results indicated significant differences in pumpkin stem thickness under each water-fertilizer coupled regulation scheme (P<0.05). The effects of irrigation and fertilization factors on pumpkin stem thickness were highly significant (P<0.01). However, the interaction between these two factors did not significantly affect pumpkin stem thickness.

**Table 3:** Effect of coupled water and fertilizer regulation on Stem thickness in pumpkin.

Year	Treatment	Stem thickness(mm)			
		seedling stage	Vine stage	Flowering stage	Maturity stage
2022	F1W1	10.15 ± 0.77c	11.08 ± 0.23c	11.44 ± 0.21e	12.34 ± 0.04d
	F1W2	11.10 ± 0.83bc	11.78 ± 0.75b	11.67 ± 0.60e	12.78 ± 0.24c
	F1W3	11.47 ± 0.84b	12.11 ± 0.26b	12.36 ± 0.33d	13.02 ± 0.01c
	F2W1	10.53 ± 0.30b	11.70 ± 0.24b	12.65 ± 0.18cd	13.10 ± 0.10c
	F2W2	11.28 ± 0.82ab	12.19 ± 0.73b	13.00 ± 0.89bc	13.53 ± 0.42b
	F2W3	11.52 ± 0.41a	12.52 ± 0.18ab	13.19 ± 0.52abc	14.26 ± 0.08a
	F3W1	11.02 ± 0.60a	11.98 ± 0.41b	13.22 ± 0.60ab	13.81 ± 0.13b
	F3W2	11.39 ± 0.33a	12.44 ± 0.36ab	13.41 ± 0.27ab	14.29 ± 0.25a
	F3W3	11.72 ± 0.23a	12.91 ± 0.26a	13.69 ± 0.29a	14.65 ± 0.24a
	CK	11.43 ± 0.20b	12.02 ± 0.27b	12.23 ± 0.32d	12.82 ± 0.43c
2023	F1W1	5.61 ± 0.23f	9.25 ± 0.39d	10.55 ± 0.17d	11.36 ± 0.21e
	F1W2	6.25 ± 0.08de	10.87 ± 0.26c	11.26 ± 0.12c	12.16 ± 0.36d
	F1W3	6.51 ± 0.03cd	10.98 ± 0.78bc	11.63 ± 0.78bc	12.54 ± 0.11cd
	F2W1	6.10 ± 0.29e	10.63 ± 0.25c	10.95 ± 0.35d	12.12 ± 0.10d
	F2W2	6.47 ± 0.43cd	11.20 ± 0.22ab	11.48 ± 0.41c	12.88 ± 0.20c
	F2W3	6.88 ± 0.42ab	11.48 ± 0.12ab	12.08 ± 0.76abc	13.51 ± 0.33b
	F3W1	6.55 ± 0.14bcd	11.22 ± 0.27ab	11.80 ± 0.69bc	12.83 ± 0.13c
	F3W2	6.82 ± 0.57abc	11.55 ± 0.30a	12.70 ± 0.04ab	13.68 ± 0.08b
	F3W3	7.03 ± 0.07a	11.65 ± 0.59a	12.40 ± 0.23a	14.34 ± 0.34a
	CK	6.40 ± 0.20de	10.86 ± 0.58c	11.59 ± 0.29bc	12.60 ± 0.20cd
Average	F1W1	7.88 ± 0.44e	10.17 ± 0.29e	10.99 ± 0.03f	11.85 ± 0.11f
	F1W2	8.67 ± 0.44d	11.33 ± 0.43cd	11.47 ± 0.29e	12.47 ± 0.10e
	F1W3	8.99 ± 0.41abc	11.55 ± 0.46cd	11.99 ± 0.47cd	12.78 ± 0.06d
	F2W1	8.32 ± 0.12de	11.16 ± 0.18d	11.80 ± 0.15de	12.61 ± 0.10de
	F2W2	8.88 ± 0.60bc	11.69 ± 0.44bc	12.24 ± 0.44bc	13.20 ± 0.16c
	F2W3	9.20 ± 0.40ab	12.00 ± 0.04ab	12.63 ± 0.45ab	13.88 ± 0.20b
	F3W1	8.78 ± 0.31bc	11.60 ± 0.23c	12.51 ± 0.45b	13.32 ± 0.13c
	F3W2	9.11 ± 0.43abc	12.00 ± 0.23ab	13.06 ± 0.13a	13.98 ± 0.09b
	F3W3	9.37 ± 0.14a	12.28 ± 0.37a	13.04 ± 0.06a	14.49 ± 0.07a
	CK	8.92 ± 0.12abc	11.44 ± 0.38cd	11.91 ± 0.28cd	12.71 ± 0.16de

Lowercase letters in the table indicate differences between different treatments in one growth stage (p<0.05).

The stem thickness gradually increased and stabilized during the flowering stage, ultimately reaching its maximum at the maturity stage. Overall, stem thickness showed a positive correlation with irrigation and fertilization factors., The stem thickness under F3W3 treatment reached its maximum at the maturity stage, significantly surpassing other treatments (P>0.05), it increased by 22.55% compared to F1W1 treatment and 23.6% compared to CK treatment.

In conclusion, improving fertilization amounts and irrigation volume promotes pumpkin stem development. Irrigation factors have a greater impact on the pumpkin stem during the early growth stage, while fertilization factors play a more significant role in the flowering stage.

#### 3.1.3 Leaf area index

**Table 4** shows the effect on pumpkin leaf area index (LAI), with consistent trends in 2022 and 2023. The results indicated that the differences in pumpkin LAI were significant (P<0.05) under each water-fertilizer coupled regulation scheme. Both irrigation and organic fertilization highly significantly influenced on pumpkin LAI (P<0.01).

**Table 4:** Effect of coupled water and fertilizer regulation on LAI.

Year	Treatment	LAI			
		seedling stage	Vine stage	Flowering stage	Maturity stage
2022	F1W1	0.117 ± 0.01ab	0.714 ± 0.01d	1.144 ± 0.04e	0.848 ± 0.01e
	F1W2	0.121 ± 0.01ab	0.741 ± 0.03d	1.198 ± 0.01d	0.899 ± 0.01e
	F1W3	0.134 ± 0.03a	0.836 ± 0.01bc	1.261 ± 0.01c	0.986 ± 0.01d
	F2W1	0.118 ± 0.01ab	0.798 ± 0.01bc	1.312 ± 0.01b	1.035 ± 0.03cd
	F2W2	0.125 ± 0.01ab	0.837 ± 0.01bc	1.379 ± 0.01a	1.123 ± 0.01ab
	F2W3	0.126 ± 0.01ab	0.892 ± 0.02a	1.391 ± 0.02a	1.146 ± 0.01a
	F3W1	0.127 ± 0.01ab	0.791 ± 0.01c	1.266 ± 0.01c	1.003 ± 0.01d
	F3W2	0.129 ± 0.01a	0.805 ± 0.03bc	1.327 ± 0.01b	1.084 ± 0bc
	F3W3	0.123 ± 0.02ab	0.825 ± 0.01bc	1.368 ± 0.01a	1.136 ± 0ab
	CK	0.109 ± 0.02b	0.849 ± 0ab	1.277 ± 0.01c	0.988 ± 0.04d
2023	F1W1	0.095 ± 0.01d	0.772 ± 0.04d	0.937 ± 0.01d	0.845 ± 0.02f
	F1W2	0.103 ± 0.01cd	0.843 ± 0.02bc	1.011 ± 0c	0.901 ± 0.01de
	F1W3	0.115 ± 0.01bc	0.891 ± 0.01ab	1.064 ± 0bc	0.983 ± 0.02c
	F2W1	0.106 ± 0.01cd	0.806 ± 0.02cd	1.069 ± 0.08bc	0.917 ± 0.03de
	F2W2	0.117 ± 0.02bc	0.893 ± 0.03ab	1.155 ± 0.01a	1.026 ± 0.01b
	F2W3	0.127 ± 0.01ab	0.929 ± 0.02a	1.181 ± 0.03a	1.078 ± 0.01a
	F3W1	0.114 ± 0.01c	0.767 ± 0.01d	1.004 ± 0.03c	0.835 ± 0.01f
	F3W2	0.128 ± 0.01ab	0.831 ± 0.01c	1.056 ± 0.01bc	0.885 ± 0.02e
	F3W3	0.133 ± 0.02a	0.851 ± 0.02bc	1.124 ± 0.01ab	0.930 ± 0.02d
	CK	0.117 ± 0.01bc	0.907 ± 0.03a	1.054 ± 0.02bc	0.937 ± 0.01d
Average	F1W1	0.106 ± 0.01c	0.740 ± 0.02e	1.041 ± 0.01e	0.846 ± 0.01e
	F1W2	0.110 ± 0.01c	0.791 ± 0.01d	1.105 ± 0.01d	0.901 ± 0.01d
	F1W3	0.124 ± 0.01ab	0.861 ± 0.01b	1.162 ± 0.01c	0.985 ± 0.01c
	F2W1	0.112 ± 0.01c	0.801 ± 0.01cd	1.191 ± 0.05b	0.976 ± 0.03c
	F2W2	0.121 ± 0.01b	0.870 ± 0.01b	1.267 ± 0a	1.074 ± 0.01ab
	F2W3	0.127 ± 0.01ab	0.911 ± 0.02a	1.286 ± 0.03a	1.112 ± 0.01a
	F3W1	0.120 ± 0.01b	0.780 ± 0.01de	1.135 ± 0.02cd	0.917 ± 0.01d
	F3W2	0.129 ± 0.01a	0.821 ± 0.02cd	1.192 ± 0.01b	0.984 ± 0.01c
	F3W3	0.128 ± 0.01a	0.841 ± 0.01bc	1.246 ± 0.01ab	1.033 ± 0.01b
	CK	0.113 ± 0.03c	0.88 ± 0.02ab	1.166 ± 0.02bc	0.963 ± 0.02c

Lowercase letters in the table indicate differences between different treatments in one growth stage (p<0.05).

Pumpkin LAI increased rapidly during the seedling stage, peaked at the flowering stage, then decreased thereafter during the maturity stage. LAI increased by 596.64% at the vine stage, 42.19% at flowering stage, compared to the previous growth stage, respectively. At maturity stage, it was reduced by 20.48% compared to the previous growth period.

Pumpkin LAI increased with irrigation volume, reaching a peak before decreasing with fertilization amounts. Under F2W3 treatment, it was the highest during flowering stage, with a 10.78% and 23.56% increase compared to the CK and F1W1 treatments, respectively. Compared to the next growth period, there was a decrease of 15.56%, 21.10%, and 22.93% for F2W3, CK, and F1W1 treatments, respectively.

In conclusion, increasing fertilization amounts and irrigation volume within a certain range promotes pumpkin LAI growth, however, excessive fertilization inhibited its growth.

### 3.2 Dry matter accumulation

The effects of water-fertilizer coupling on pumpkin dry matter accumulation in 2022 and 2023 remained basically the same (**Table 5**). The dry matter accumulation of pumpkin showed significant differences under each water-fertilizer coupled regulation scheme during the same growth period (P<0.05). Irrigation level and organic fertilization amount had highly significant effect on dry matter accumulation of pumpkin rhizomes and leaves (P<0.01).

**Table 5:** Effect of coupled water and fertilizer regulation on dry matter accumulation in pumpkin.

Year	Treatment	Rhizome fresh weight	Rhizome dry weight	Rhizome dry rate	Leaf fresh weight	Leaf dry weight	Leaf dry rate
2022	F1W1	941 ± 61.74d	100.40 ± 10.00e	0.107	349.61 ± 11.72d	102.31 ± 9.68d	0.293
	F1W2	1155.48 ± 30.02c	110.63 ± 8.72d	0.096	360.66 ± 26.37cd	110.67 ± 16.91cd	0.307
	F1W3	1159.58 ± 28.13c	124.66 ± 5.29bc	0.108	379.60 ± 17.69bcd	119.99 ± 5.63bcd	0.316
	F2W1	1152.74 ± 15.43c	118.59 ± 6.56cd	0.103	410.25 ± 8.87abc	124.31 ± 1.46bc	0.303
	F2W2	1190.67 ± 5.30bc	124.05 ± 8.72bc	0.104	438.24 ± 13.14a	137.67 ± 2.15ab	0.314
	F2W3	1227.87 ± 24.68bc	131.36 ± 1.01ab	0.107	447.17 ± 24.63a	144.57 ± 1.78a	0.323
	F3W1	1238.19 ± 32.83b	128.92 ± 2.89ab	0.104	396.56 ± 35.90abcd	112.08 ± 5.67cd	0.283
	F3W2	1268.23 ± 13.29ab	133.30 ± 9.07a	0.105	419.08 ± 20.49ab	122.87 ± 4.79bc	0.293
	F3W3	1335.30 ± 21.23a	134.03 ± 7.23a	0.100	436.03 ± 17.02a	129.16 ± 4.95abc	0.296
	CK	1150.27 ± 30.98c	116.70 ± 9.69cd	0.101	376.00 ± 11.84bcd	126.43 ± 11.40abc	0.336
2023	F1W1	823.23 ± 25.50b	73.95 ± 1.90e	0.09	261.13 ± 9.16c	77.76 ± 3.47e	0.298
	F1W2	837.73 ± 34.62b	79.10 ± 6.66de	0.094	281.37 ± 81.70bc	84.15 ± 7.10e	0.299
	F1W3	853.63 ± 38.80ab	82.75 ± 1.74cde	0.097	290.43 ± 41.09abc	87.54 ± 1.90de	0.301
	F2W1	844.43 ± 37.38ab	83.83 ± 2.77cde	0.099	307.67 ± 22.02abc	91.33 ± 1.30cd	0.297
	F2W2	858.13 ± 58.33ab	89.99 ± 5.84bc	0.105	325.30 ± 6.20ab	103.73 ± 1.09b	0.319
	F2W3	876.2 ± 21.68ab	91.71 ± 0.67bc	0.105	346.90 ± 22.31a	113.71 ± 4.27a	0.328
	F3W1	863.73 ± 43.02ab	89.38 ± 1.90bcd	0.103	306.40 ± 2.80abc	87.65 ± 5.94de	0.286
	F3W2	885.80 ± 19.60ab	95.53 ± 0.81ab	0.108	321.71 ± 17.76abc	94.17 ± 1.68bcd	0.293
	F3W3	940.57 ± 28.30ab	102.59 ± 3.19a	0.109	332.03 ± 3.61ab	102.05 ± 1.22bc	0.307
	CK	835.27 ± 24.71b	82.07 ± 6.43cde	0.098	308.00 ± 10.31abc	99.58 ± 6.87bc	0.323
Average	F1W1	882.12 ± 42.11e	87.17 ± 4.37f	0.099	305.37 ± 3.22d	90.04 ± 6.36e	0.295
	F1W2	996.61 ± 4.44d	94.87 ± 1.04ef	0.095	321.01 ± 32.61cd	97.41 ± 10.94de	0.303
	F1W3	1006.61 ± 30.99d	103.71 ± 2.11cd	0.103	335.02 ± 22.47cd	103.77 ± 3.58cd	0.310
	F2W1	998.59 ± 21.55d	101.21 ± 3.79de	0.101	358.96 ± 8.63abc	107.82 ± 0.41cd	0.300
	F2W2	1024.40 ± 27.31cd	107.02 ± 6.46bcd	0.104	381.77 ± 3.55ab	120.70 ± 0.53ab	0.316
	F2W3	1052.03 ± 2.20bc	111.53 ± 0.30abc	0.106	397.03 ± 14.72a	129.14 ± 2.03a	0.325
	F3W1	1050.96 ± 37.86bc	109.15 ± 2.05bc	0.104	351.48 ± 18.80bc	99.87 ± 5.71de	0.284
	F3W2	1077.02 ± 3.50b	114.42 ± 4.23ab	0.106	370.39 ± 9.74ab	108.52 ± 3.04bcd	0.293
	F3W3	1137.93 ± 19.29a	118.31 ± 4.79a	0.104	384.03 ± 9.31ab	115.61 ± 2.73bc	0.301
	CK	992.77 ± 21.68d	99.39 ± 8.05de	0.100	342.00 ± 9.80c	113.01 ± 2.46bc	0.330

Lowercase letters in the table indicate differences between treatments (p<0.05).

In 2022 and 2023, the fresh weight of pumpkin rhizomes showed a positive correlation with irrigation and organic fertilization. The largest rhizomes fresh weight was achieved under F3W3 treatment, which was 29.9% higher than that under the F1W1 treatment, 14.56% higher than that under the CK, respectively. As a whole, the dry weight of pumpkin rhizomes showed an increasing trend with increasing irrigation volume and organic fertilization amounts, reaching a maximum under F3W3 treatment, which was 36.15% higher than the F1W1 treatment, and 18.91% higher than the CK treatment. Pumpkin rhizomes drying rate was the highest under F2W3 treatment.

The fresh weight of pumpkin leaves exhibited a positive correlation with the irrigation volume, while it displayed an increasing-then-decreasing pattern in response to the organic fertilization amount. Leaf fresh weight reached a maximum under F2W3 treatment, which had increased by 30.00% compared to the lowest F1W1 treatment, and increased by 16.07% compared to CK treatment. The pumpkin leaf dry weight overall increased with increasing irrigation volume, also showed an increasing and then decreasing trend with increasing fertilization amount. Reaching the maximum under F2W3 treatment, which having been 43.43% and 14.18% higher than the lowest treatments F1W1 and CK, respectively. Leaf drying rate achieved its maximum under CK and F2W3 treatment was next.

The leaf drying rate of pumpkin was positively correlated with irrigation volume. It showed an increasing and then decreasing trend with fertilizer application amount. These indicate that increasing irrigation volume and fertilization amount promote dry matter accumulation in leaves, although excessive fertilization amount has an inhibitory effect.

### 3.3 Pumpkin yield

The effect trend of pumpkin yield remained consistent in both 2022 and 2023 year (**Table 6**). The yield varied significantly across treatments (P<0.01),and significantly affected by irrigation, fertilization and their interaction(P<0.01).

**Table 6:** Effect of coupled water and fertilizer regulation on pumpkin yield.

Treatment	Yield(kg/ha)		
	2022	2023	Average
F1W1	22030.15 ± 105.41g	21605.14 ± 406.16f	21817.65 ± 163.82e
F1W2	23628.75 ± 183.37f	24131.48 ± 421.46e	23880.12 ± 256.47d
F1W3	24914.16 ± 131.15d	26705.73 ± 566.08bc	25809.94 ± 242.56c
F2W1	24225.80 ± 133.73e	24632.31 ± 353.09e	24429.06 ± 243.09d
F2W2	26992.70 ± 168.01b	27508.7 ± 526.77b	27250.7 ± 326.17b
F2W3	30726.13 ± 207.54a	31476.13 ± 207.54a	31101.13 ± 207.54a
F3W1	24635.06 ± 181.92d	24885.06 ± 181.92de	24760.06 ± 181.92d
F3W2	25713.12 ± 263.11c	26463.12 ± 263.11bcd	26088.12 ± 263.11c
F3W3	26044.72 ± 167.67c	26794.72 ± 167.67bc	26419.72 ± 167.67c
CK	24119.46 ± 160.08e	25258.67 ± 644.52cde	24689.06 ± 340.74d

Lowercase letters in the table indicate differences between treatments (p<0.05).

**Figure 2:**
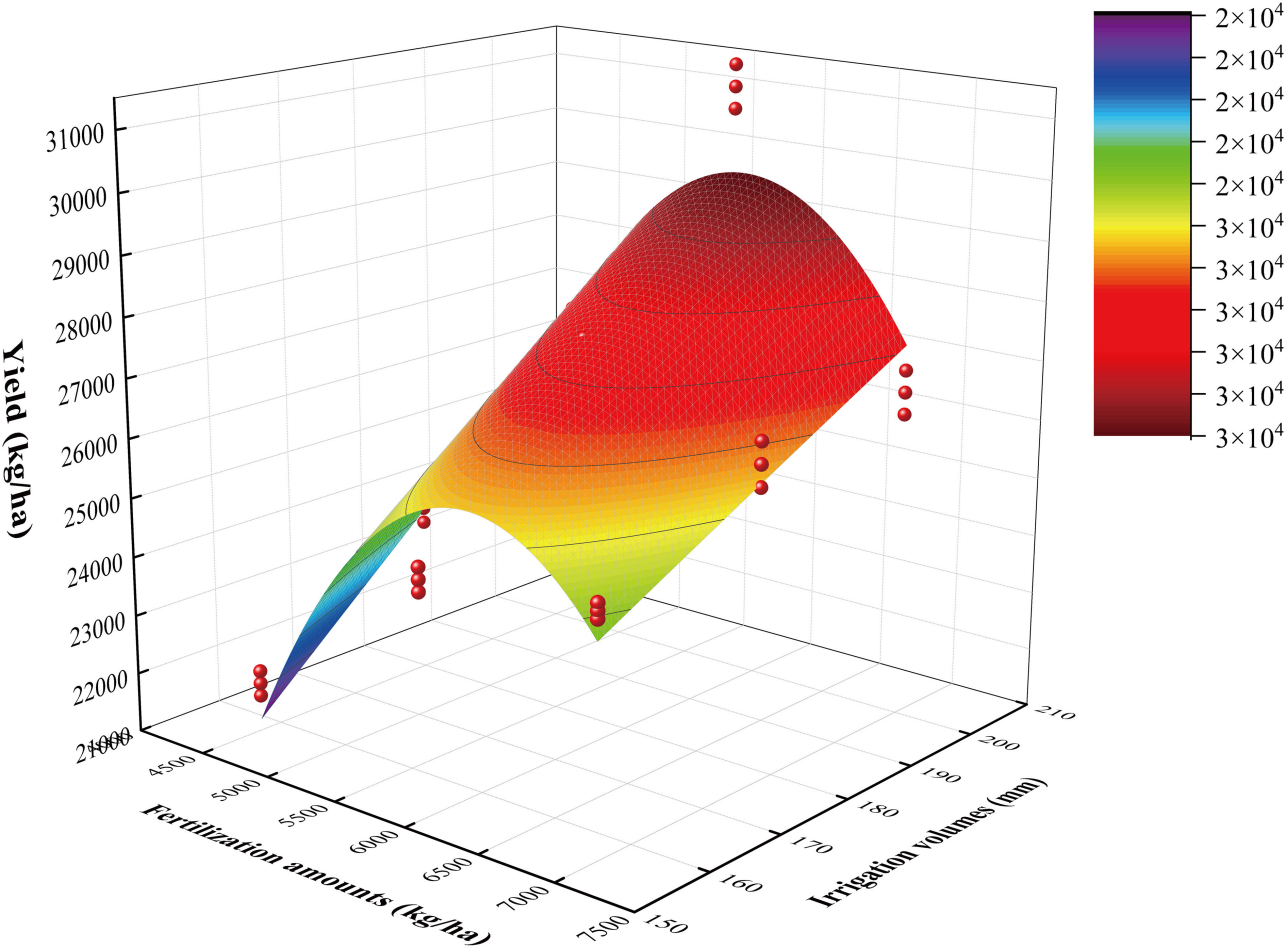
Regression model of pumpkin yield with irrigation and fertilization coupled.

Pumpkin yield was positively correlated with irrigation volume and exhibited a trend of initially increasing and then decreasing with the increase of fertilization amount. It reached the maximum under F2W3 treatment, significantly higher than other treatments, increasing by 25.70% and 42.55%, compared with CK and F1W1 with the lowest yield, respectively. The yield did not significantly increase with increasing irrigation volume under high fertilization amount (F3). When the amount of organic fertilization was raised from F2 to F3, there was a decline in yield. Pumpkin yields were ranked asW2>W3>W1 in terms of irrigation factors, F2>F3>F1 by organic fertilization amount. A regression model was constructed, and a surface was fitted using the average data of 2022 and 2023 years, with irrigation volume and organic fertilization amounts as independent variables, and pumpkin yield as dependent variable (**Figure 2**). The F-value for the model was 27.81, p<0.01, indicating that the model was extremely significant. Furthermore, the R^2^ value of 0. 877 indicated a good model fit for the model. The regression model illustrated that increasing the level of irrigation and organic fertilization amounts was beneficial for pumpkin to obtain high yield, but it began to decline when reaching the critical point. All those further illustrated that theF2W3 treatment could promote high yield of pumpkin, while the low irrigation volume and organic fertilizer amount, along with the high amount of organic fertilizer, exerted an inhibitory effect on pumpkin yield formation.

### 3.4 Evapotranspiration (ET)

The effect of different water-fertilizer coupled regulation on evapotranspiration (ET) in 2022 and 2023 is shown in **Table 7**. Significant differences in ET were observed among treatments at each growth stage (P<0.05). Irrigation factors significantly influenced ET, while the impact of fertilization factors varied across growth stages, with no significance effect during the vine stage (P<0.05) and extreme significance in other stages (P<0.01). However, their interaction on ET varies with the growth period.

**Table 7:** Effect of coupled water and fertilizer regulation on ET.

Year	Treatment	ET(mm)				
		seedling stage	Vine stage	Flowering stage	Maturity stage	Total
2022	F1W1	23.48 ± 0.80e	84.90 ± 3.47d	147.26 ± 2.78d	41.61 ± 1.42e	297.25 ± 8.20e
	F1W2	24.71 ± 0.83e	94.16 ± 2.03abc	154.21 ± 2.12c	47.48 ± 1.12d	320.56 ± 11.25d
	F1W3	35.33 ± 0.70bcd	98.83 ± 1.12a	159.57 ± 2.30ab	50.79 ± 1.04c	344.53 ± 5.99bc
	F2W1	24.51 ± 0.40e	85.34 ± 1.16d	148.93 ± 2.10d	45.60 ± 1.28d	304.41 ± 11.15e
	F2W2	31.37 ± 1.00d	90.37 ± 3.02bcd	155.95 ± 1.97bc	51.70 ± 1.63c	329.38 ± 6.30cd
	F2W3	38.35 ± 1.60ab	96.74 ± 1.07ab	161.05 ± 2.47ab	60.22 ± 1.36b	356.36 ± 12.40ab
	F3W1	26.13 ± 2.80e	89.51 ± 1.33bcd	151.91 ± 2.36cd	50.65 ± 1.65c	318.19 ± 2.30d
	F3W2	32.29 ± 3.80cd	91.89 ± 5.98abcd	156.90 ± 2.45bc	59.17 ± 1.18b	340.25 ± 1.12bc
	F3W3	41.12 ± 1.30a	91.85 ± 5.91abcd	163.63 ± 2.26a	71.12 ± 1.63a	367.71 ± 3.80a
	CK	36.06 ± 1.70bc	89.15 ± 6.78d	160.35 ± 1.90ab	60.21 ± 1.37b	345.76 ± 4.30bc
2023	F1W1	22.51 ± 0.70e	78.89 ± 3.65bc	105.94 ± 2.38d	37.56 ± 1.12e	244.97 ± 9.54f
	F1W2	25.15 ± 0.63e	86.75 ± 5.33ab	125.08 ± 2.22abc	41.50 ± 1.52d	278.70 ± 3.40cd
	F1W3	34.78 ± 0.75bc	91.53 ± 4.53a	129.49 ± 2.40ab	48.47 ± 1.44c	303.86 ± 3.78b
	F2W1	23.12 ± 0.65e	79.19 ± 3.26bc	119.61 ± 2.70c	37.88 ± 1.78e	259.49 ± 0.54e
	F2W2	32.69 ± 0.90cd	83.01 ± 5.88abc	125.80 ± 1.67ab	47.59 ± 1.68c	289.06 ± 2.82c
	F2W3	39.63 ± 3.40ab	89.45 ± 7.67ab	129.96 ± 2.27ab	53.69 ± 1.23b	312.57 ± 4.79ab
	F3W1	27.52 ± 0.70de	74.62 ± 6.73c	124.55 ± 2.22bc	43.58 ± 1.69d	270.44 ± 0.53de
	F3W2	33.61 ± 1.68c	84.54 ± 6.58abc	127.16 ± 2.65ab	56.52 ± 1.75b	301.50 ± 0.42b
	F3W3	42.38 ± 2.63a	88.26 ± 5.30ab	131.26 ± 2.76a	61.44 ± 1.49a	323.56 ± 1.92a
	CK	37.47 ± 2.80abc	81.68 ± 6.28abc	128.80 ± 2.20ab	55.93 ± 1.33b	303.67 ± 1.16b
Average	F1W1	23.00 ± 0.75e	81.89 ± 3.56c	126.60 ± 2.58f	39.59 ± 1.27e	271.11 ± 8.87f
	F1W2	24.93 ± 0.73e	90.46 ± 3.68abc	139.64 ± 2.17cd	44.49 ± 1.32d	299.63 ± 3.93de
	F1W3	35.06 ± 0.73cd	95.18 ± 2.83a	144.53 ± 2.35ab	49.63 ± 1.24c	324.20 ± 4.88bc
	F2W1	23.82 ± 0.53e	82.27 ± 2.21c	134.27 ± 2.40e	41.74 ± 1.53e	281.95 ± 5.81f
	F2W2	32.03 ± 0.95d	86.69 ± 4.45abc	140.88 ± 1.82cd	49.64 ± 1.65c	309.22 ± 1.74d
	F2W3	38.99 ± 2.50ab	93.10 ± 4.37ab	145.51 ± 2.37ab	56.96 ± 1.30b	334.46 ± 8.60ab
	F3W1	26.82 ± 1.75e	82.07 ± 4.03c	138.23 ± 2.29d	47.11 ± 1.67c	294.31 ± 1.41e
	F3W2	32.95 ± 2.74d	88.21 ± 6.28abc	142.03 ± 2.55bc	57.85 ± 1.47b	320.87 ± 0.44c
	F3W3	41.75 ± 1.97a	90.06 ± 5.60abc	147.45 ± 2.51a	66.28 ± 1.56a	345.63 ± 2.86a
	CK	36.77 ± 2.25bc	85.41 ± 6.53bc	144.57 ± 2.05ab	58.07 ± 1.35b	324.72 ± 2.73bc

Lowercase letters in the table indicate differences between different treatments in one growth stage (p<0.05).

The irrigation factors were the main determinants of ET. The highest ET of pumpkin was observed under F3W3 treatment, while the lowest under the F1W1 treatment. The data clearly showed a positive correlation between ET and irrigation volume, organic fertilizer amount. It initially increased and then decreased during the period, with consistent both 2022 and 2023.

The flowering stage was critical for pumpkin water demand, with the highest ET throughout its growth,. The proportion of ET during this stage was 42.66%, 46.70%, and 44.52% for F3W3, F1W1, and CK treatments, respectively, in relation to the total ET. The total ET of F3W3 increased by 27.48% and 2.65%, respectively, compared to F1W1 and CK. In conclusion, appropriately reducing the irrigation level and organic fertilizer amount could reduce ineffective ET. The application of organic fertilizer increased ET during pumpkin growth.

### 3.5 Water and fertilizer use efficiency

The effects of coupled water-fertilizer regulation on WUE, IWUE and PFP of pumpkin in 2022 and 2023 is shown in **Table 8**, the basically consistent trends of the two years being. The results showed that water-fertilizer coupling significantly affected the WUE, IWUE and PFP for pumpkin (P<0.05).

**Table 8:** Effect of coupled water and fertilizer regulation on water and fertilizer utilization efficiency of pumpkin.

Year	Treatment	ET (mm)	WUE (kg·m^-3^)	IWUE (kg·m^-3^)	PFP
2022	F1W1	297.25 ± 8.20e	7.41 ± 0.04f	13.99 ± 0.07c	4.90 ± 0.02e
	F1W2	320.56 ± 11.25d	7.37 ± 0.06f	13.13 ± 0.10d	5.25 ± 0.04d
	F1W3	344.53 ± 5.99bc	7.23 ± 0.04g	12.30 ± 0.06e	5.54 ± 0.03b
	F2W1	304.41 ± 11.15e	7.96 ± 0.04c	15.38 ± 0.08a	4.25 ± 0.02g
	F2W2	329.38 ± 6.30cd	8.19 ± 0.05b	15.00 ± 0.09b	4.74 ± 0.03f
	F2W3	356.36 ± 12.40ab	8.62 ± 0.06a	15.17 ± 0.10b	5.39 ± 0.04c
	F3W1	318.19 ± 2.30d	7.74 ± 0.06d	15.64 ± 0.12a	3.57 ± 0.03i
	F3W2	340.25 ± 1.12bc	7.56 ± 0.08e	14.29 ± 0.15c	3.73 ± 0.04h
	F3W3	367.71 ± 3.80a	7.08 ± 0.05h	12.86 ± 0.08d	3.77 ± 0.02h
	CK	345.76 ± 4.30bc	6.98 ± 0.05h	11.91 ± 0.08f	17.87 ± 0.12a
2023	F1W1	244.97 ± 9.54f	8.82 ± 0.46cd	13.72 ± 0.72c	4.80 ± 0.25cd
	F1W2	278.70 ± 3.40cd	8.66 ± 0.48cd	13.41 ± 0.75c	5.36 ± 0.3bc
	F1W3	303.86 ± 3.78b	8.79 ± 0.47cd	13.19 ± 0.71cd	5.93 ± 0.32b
	F2W1	259.49 ± 0.54e	9.49 ± 0.58ab	15.64 ± 0.95ab	4.32 ± 0.26de
	F2W2	289.06 ± 2.82c	9.60 ± 0.67ab	15.42 ± 1.08ab	4.87 ± 0.34cd
	F2W3	312.57 ± 4.79ab	10.07 ± 0.07a	15.54 ± 0.10a	5.52 ± 0.04bc
	F3W1	270.44 ± 0.53de	9.20 ± 0.07bc	15.80 ± 0.12a	3.61 ± 0.03e
	F3W2	301.50 ± 0.42b	8.78 ± 0.09cd	14.70 ± 0.15b	3.84 ± 0.04e
	F3W3	323.56 ± 1.92a	8.28 ± 0.05d	13.23 ± 0.08cd	3.88 ± 0.02e
	CK	303.67 ± 1.16b	8.32 ± 0.45d	12.47 ± 0.68d	18.71 ± 1.01a
Average	F1W1	271.11 ± 8.87f	8.05 ± 0.20d	13.85 ± 0.35c	4.85 ± 0.12d
	F1W2	299.63 ± 3.93de	7.97 ± 0.25d	13.27 ± 0.42d	5.31 ± 0.17c
	F1W3	324.20 ± 4.88bc	7.96 ± 0.24d	12.75 ± 0.38d	5.74 ± 0.17b
	F2W1	281.95 ± 5.81f	8.66 ± 0.28bc	15.51 ± 0.50a	4.29 ± 0.14e
	F2W2	309.22 ± 1.74d	8.85 ± 0.30b	15.21 ± 0.52a	4.80 ± 0.16d
	F2W3	334.46 ± 8.60ab	9.30 ± 0.06a	15.36 ± 0.10a	5.46 ± 0.04bc
	F3W1	294.31 ± 1.41e	8.41 ± 0.06c	15.72 ± 0.12a	3.59 ± 0.03f
	F3W2	320.87 ± 0.44c	8.13 ± 0.08d	14.49 ± 0.15b	3.78 ± 0.04f
	F3W3	345.63 ± 2.86a	7.64 ± 0.05e	13.05 ± 0.08d	3.83 ± 0.02f
	CK	324.72 ± 2.73bc	7.60 ± 0.20e	12.19 ± 0.33e	18.29 ± 0.49a

Lowercase letters in the table indicate differences between treatments (p<0.05).

The WUE exhibited a positive correlation with increasing irrigation volume under F2 levels, while it initially decreased with increasing irrigation volume under the F1, F3 levels. The WUE influenced by irrigation volume in the order of W1>W2>W3, and under the influence of fertilization amounts is F2>F3>F1. The F2W3 treatment reached the highest value, significantly superior to the other treatments, and showing an increase of 18.45% and 25.84% compared to CK and F1W1 treatment, respectively. The summary is that modest increasing irrigation volume enhances WUE, while moderate fertilization amounts increase it but excessive amounts inhibit it.

The IWUE showed a negative correlation with irrigation volume and exhibited an initial increase followed by a subsequent decrease with increasing fertilization amount. The IWUE was highest with F3W1 treatment, increasing by 28.96% and 13.62% compared to CK and F1W1 treatment, respectively. The order of IWUE was F2>F3>F1 under different fertilization amounts, and it was W1>W2>W3 under different irrigation volume. Thus, reducing irrigation volume and fertilization amounts appropriately can enhance IWUE. The PFP showed a positive correlation with irrigation volume and a negative correlation with fertilization amount. The maximum PFP was achieved under the F1W3 treatment, showing an 18.35% increase compared to F1W1 treatment, the percentage is 68.65% decrease compared to CK treatments.

### 3.6 Comprehensive evaluation of pumpkin water-fertilizer coupling scheme

#### 3.6.1 Correlation analysis

**Figure 3:**
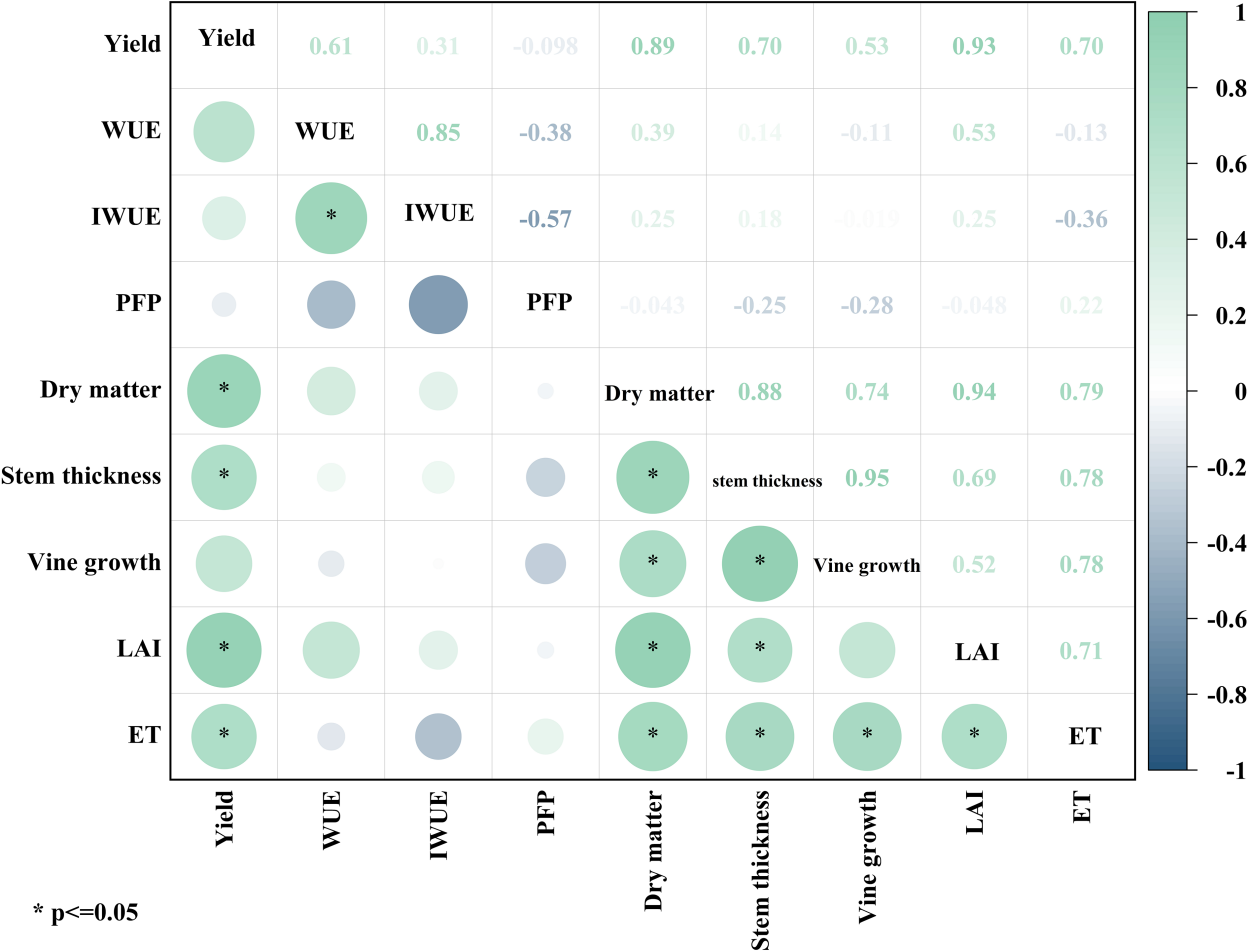
Correlation analysis.

Correlation analysis based on nine indicators of pumpkin growth index, yield, ET, WUE, IWUE and PFP under each water-fertilizer coupling scheme (**Figure 3**) showed that yield was significantly and positively correlated with dry matter, stem thickness, LAI and ET (P<0.05), with the correlation coefficients of 0.89, 0.70, 0.93 and 0.70, respectively. WUE was significantly and positively correlated with IWUE, the correlation coefficient is 0.85. Total dry matter accumulation was significantly and positively correlated with stem thickness, vine length, LAI and ET with correlation coefficients of 0.88, 0.74, 0.94, and 0.79, respectively. Stem thickness was significantly and positively correlated with vine length, LAI and ET with correlation coefficients of 0.95, 0.69 and 0.78, respectively. Vine length was significantly and positively correlated with ET, the correlation coefficient is 0.78.

#### 3.6.2 Principal component analysis (PCA)

Principal component analysis (PCA) method was performed based on nine indicators of pumpkin under each water-fertilizer coupling treatment using SPSS software, the results being shown in **Table 9**. Taking the average value of 2022 and 2023 years as an example, at first, the principal components would be selected. In here, the first 3 principal components, with eigenvalues > 1 and the cumulative contribution rate with 95.63% in the extraction results, were selected, all which indicated that these 3 principal components had been able to represent most of the information of the indexes measured and meet the requirements of the PCA. Among them, the contribution rate was 55.77% of the first principal component, and 26.91% of the second principal component, and 12.95% of the third principal component, respectively. Then, for the three principal components analyzed, a matrix of component scores was calculated. Finally, a linear relationship was obtained according to the principal component model, as follows.


$$\begin{array}{l} F_{1}=0.184X_{1}+0.083X_{2}+0.054X_{3}-0.038X_{4}+0.195X_{5}+0.182X_{6}\\ \;+0.157X_{7}+0.182X_{8}+0.159X_{9} \end{array}$$

$$\begin{array}{l} F_{2}=0.058X_{1}+0.350X_{2}+0.378X_{3}-0.250X_{4}-0.019X_{5}-0.066X_{6}\\ \;-0.136X_{7}+0.031X_{8}-0.240X_{9} \end{array}$$

$$\begin{array}{l} F_{3}=0.247X_{1}+0.270X_{2}-0.072X_{3}+0.605X_{4}+0.100X_{5}\\ \;-0.280X_{6}-0.434X_{7}+0.269X_{8}+0.061X_{9} \end{array}$$

$$\begin{array}{l} F=\left(0.558/0.956\right)F_{1}+\left(0.269/0.956\right)F_{2}+\left(0.129/0.956\right)F_{3} \end{array}$$

**Table 9:** Load matrix, eigenvalues, contribution rate and weights of each principal component factor.

Indicators		PCA1			PCA2			PCA3		
		2022	2023	Average	2022	2023	Average	2022	2023	Average
Factor loading	Yield	0.90	0.92	0.92	-0.10	0.24	0.14	0.33	0.26	0.29
	WUE	0.50	0.26	0.42	-0.77	0.95	0.85	0.38	0.17	0.32
	IWUE	0.31	0.20	0.27	-0.91	0.89	0.92	0.01	-0.27	-0.08
	PFP	-0.28	-0.09	-0.19	0.62	-0.52	-0.61	0.67	0.73	0.71
	Dry matter accumulation	0.96	0.96	0.98	0.12	0.02	-0.05	0.20	-0.03	0.12
	Stem thickness	0.93	0.91	0.91	0.06	-0.24	-0.16	-0.30	-0.31	-0.33
	Vine length	0.85	0.71	0.79	0.31	-0.42	-0.33	-0.41	-0.56	-0.51
	LAI	0.96	0.69	0.92	0.02	0.27	0.08	0.10	0.60	0.31
	ET	0.73	0.87	0.80	0.66	-0.45	-0.58	0.05	0.15	0.07
Eigenvalues		5.18	4.46	5.02	2.38	2.52	2.24	1.01	1.50	1.17
Contribution rate(%)		57.58	49.55	55.77	26.44	28.01	26.91	11.25	16.64	12.95
Cumulative contribution(%)		57.58	49.55	55.77	84.01	77.56	82.68	95.27	94.21	95.63
weights(%)		60.44	52.60	58.32	27.75	29.74	28.14	11.81	17.67	13.54

Where, *X*_1_ to *X*_9_ respectively represented yield, WUE, IWUE, PFP, dry matter accumulation, stem thickness, vine length, LAI and ET; *F*_1_, *F*_2_ and *F*_3_ represented the score of the three principal component, respectively; and *F* represented the sum of the three principal component scores.

The results of **Table 9** showed that the variance contribution rate of PCA1 the maximum value of the three principal components, indicating that it had the greatest influence on the comprehensive evaluation. The main influencing factors of PCA1 were yield, dry matter, stem thickness, LAI and ET, which were positively correlated with PCA1, indicating the larger value of the principal component loading, the larger the five indexes were. The main influence factors of PCA2 were WUE and IWUE, being positively correlated with PCA2, and indicating that the larger principal component loading value, the larger these 2 indicators were. The PCA3 variance contribution accounted for 15.44%, with PFP identified as the primary influencing factor exhibiting a positive correlation with PCA3. This suggests that higher values of principal component loading correspond to larger PFP values.

The comprehensive evaluation results of different water-fertilizer coupled regulation schemes were obtained by calculating comprehensive scores and ranking them (**Table 10**). The higher composite score of PCA, the better the growth, yield, and water fertilizer utilization of pumpkin. The composite scores were positive, indicating that the growth, yield, and water-fertilizer utilization of pumpkin were higher than the mean value. Six of the all coupled water-fertilizer regulation schemes, F2W1, F2W2, F2W3, F3W1,F3W2, and F3W3, were higher than the mean value; while F1W1, F1W2, F1W3 and CK were lower than the mean value. The composite score values of the 10 water-fertilizer coupling regulation schemes ranged from -0.95 to 1.33, indicating that the comprehensive growth of pumpkin under different water-fertilizer coupling regulation schemes varied significantly. According to the comprehensive score, the comprehensive ranking of each treatment had been gotten. Moreover, the comprehensive score under F2W3 treatment was the 1st, indicating that the treatment was relatively better, and the 2022 and 2023 data remained consistent. The F2W3 treatment demonstrated superior efficacy in promoting pumpkin growth, enhancing yield, and optimizing water and fertilizer utilization.

**Table 10:** Comprehensive scores of treatments under different water-fertilizer coupling regulation.

Treatment	PCA1			PCA2			PCA3			Aggregate score			Rank		
	2022	2023	Average	2022	2023	Average	2022	2023	Average	2022	2023	Average	2022	2023	Average
F1W1	-1.695	-1.793	-1.763	-0.675	0.134	0.435	-0.344	-0.215	-0.315	-1.252	-0.941	-0.948	10	10	10
F1W2	-0.987	-0.827	-0.929	0.049	-0.324	-0.196	-0.332	0.054	-0.242	-0.622	-0.522	-0.629	9	8	9
F1W3	-0.26	0	-0.12	0.837	-0.419	-0.682	-0.274	0.588	0.062	0.043	-0.021	-0.254	6	5	7
F2W1	-0.336	-0.633	-0.453	-1.174	1.124	1.185	0.253	-0.099	0.212	-0.499	-0.016	0.098	8	7	4
F2W2	0.599	0.528	0.603	-0.756	1.009	0.865	0.741	0.531	0.703	0.240	0.671	0.690	4	2	2
F2W3	1.568	1.56	1.597	-0.619	1.22	0.856	1.33	0.950	1.152	0.933	1.351	1.328	1	1	1
F3W1	0.042	-0.338	-0.157	-0.823	0.567	0.767	-0.715	-1.221	-0.906	-0.288	-0.225	0.002	7	9	6
F3W2	0.682	0.498	0.578	0.099	-0.309	-0.16	-0.899	-1.064	-0.981	0.334	-0.018	0.159	3	6	3
F3W3	1.091	1.251	1.138	1.267	-1.432	-1.319	-1.456	-1.291	-1.445	0.839	0.004	0.096	2	4	5
CK	-0.705	-0.246	-0.494	1.793	-1.57	-1.751	1.696	1.767	1.761	0.272	-0.284	-0.542	5	3	8

#### 3.6.3 Cluster analysis

**Figure 4:**
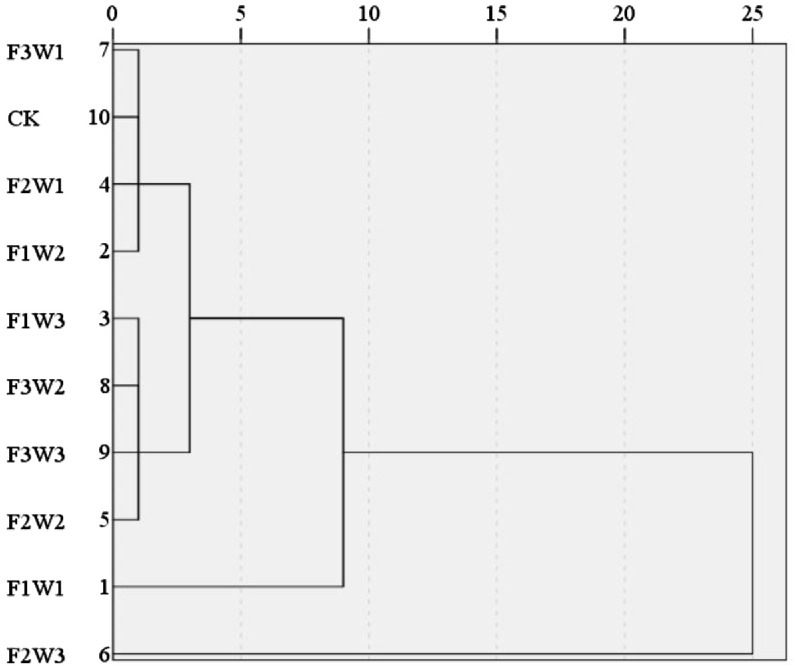
Cluster analysis.

Based on the nine indicators that could reflect the growth, yield, and efficiency aspects of pumpkin, a systematic cluster analysis was using SPSS 27 and a horizontal spectrum was drawn (**Figure 4**). The 10 treatments at a Euclidean distance of 2.5 were categorized into four groups, the first being F2W3, the second including F2W2, F3W3, F3W2 and F1W3, the third including F1W2, F2W1, CK and F3W1, and the fourth including F1W1. In the first category, values of several indexes reached the maximum, such as yield, WUE, dry matter and LAI, the remaining indicators remain at a significantly elevated level, of which the principal component score was the 1st. In the second category, indicators reached higher levels, with principal component scores all being in the top 5. In the third and fourth categories, the indicators were observed to be at a comparatively lower level, with all of the principal component scores ranking toward the bottom of the list. The results of the cluster analysis were found to be largely consistent with those obtained from the PCA, and the data remained consistent between the 2022 and 2023.

## 4 Discussion

In actual agricultural production, the growth and development of pumpkins require appropriate water and fertilizer conditions due to the complex interactions between them. The results indicated highly significant responses of irrigation and fertilization factors on pumpkin vine length, stem thickness and leaf area index, and dry matter (P<0.01). In addition, Within a certain range, increasing fertilization application amount and irrigation quota could significantly promote the growth of pumpkin plants, which was consistent with the findings of Cheng (Cheng, 2020). The coupled regulation of water-fertilizer could significantly promote the leaves growth, dry matter accumulation of pumpkin (Cheng et al., 2019), the same rule was also found in the study of watermelon (Hong et al., 2022). The results of this study indicated that either too high or too low water and fertilizer levels would have negative coupling effect, thereby affecting pumpkin leaf growth. Under low water and fertilizer conditions, insufficient soil moisture led to reduce free water and total water content in pumpkin leaves. Consequently, this hindered the leaves growth and dry matter accumulation of pumpkin, aligning with Khalili’s findings (Khalili and Nejatzadeh, 2021). In this study, higher fertilizer amount inhibited the growth of pumpkin leaf area. The leaf area index (LAI) of pumpkin initially increased and then decreased with increasing fertilization level, consistent with the findings of Naderi (Naderi et al., 2017), as well as a similar pattern was observed in studies on green vegetables and greenhouse tomatoes (Fan et al., 2017; Yue et al., 2022). The excessive nutrition, resulting from high levels of irrigation and fertilization, leaded to futile growth, caused an increase in the vegetative parts of the pumpkin plants, subsequently in turn affected yield formation. This decline in pumpkin yields may be attributed to the excessive use of nutrients and water during the early stages, as well as a deficiency of nutrients for pumpkin fruits during the later stages. These findings align with Ma’s conclusions (Ma et al., 2023), and are similar to those drawn by numerous scholars studying various crops such as sugarcane (Wu et al., 2022), pear (Wang et al., 2022a) and potato (Zhang et al., 2023a).

The conclusion indicated that irrigation, fertilization, and their interaction had a significant impact on yield (P<0.01). Based on experimental data in 2022 and 2023, the yield increased with higher irrigation volume, while the effect of fertilization amounts showed an initial increase followed by a decrease. The excessive fertilization negatively impacted the water-fertilizer coupling during pumpkin yield formation stage, thereby impeding yield development. The amount of fertilizer applied has a greater influence than the amount of irrigation, and appropriate irrigation and fertilization scheme could enhance pumpkin yield (Budak and Güneş, 2023). The increase in irrigation volume under high fertilization amount did not significantly enhance pumpkin yield, consistent with Khalili (Khalili and Nejatzadeh, 2021). The yield initially increases and then decreases with the improvement of fertilizer amounts and irrigation amounts under their coupled control. The relationship between crop yield and irrigation volume and fertilizer amounts follows a quadratic parabola. Excessive or insufficient irrigation and fertilizer levels negatively impact crop yield (Xiao et al., 2021; Zhang et al., 2023b). This conclusion is slightly different from the experimental results. The pumpkin yield did not decrease with the increase of irrigation volume, which may be attributed to the fact that the irrigation volume designed in this experiment did not reach the gradient of negative impact on yield. The experiment revealed a threshold for the impact of water-fertilizer coupling regulation on pumpkin yield. When fertilization amounts exceeded this threshold, a negative effect of water-fertilizer coupling emerged, further increasing the fertilization amounts inhibited the formation of pumpkin yield, which aligns with previous findings (Chen et al., 2019). Therefore, the coupling regulation of water and fertilizer in pumpkin must be carried out within an appropriate range, consistent with the “dilution effect” conclusion by Wang (Wang et al., 2015). Similar findings have also been observed in studies on wolfberry (Liu et al., 2021a), Panax notoginseng (Liu et al., 2021b), and summer maize (Ma et al., 2021).

This study found that irrigation factors significantly influenced Evapotranspiration (ET) (P<0.01). The impact of fertilization factors on ET varied with the growth stage of pumpkins but remained significant overall (P<0.01). The interaction between the two factors on ET changes with the growth period, and the overall level is significant, and irrigation factors had a greater influence than fertilization factors. The water demand of pumpkin varied throughout its growth period in this experiment. The rate of increase in ET was relatively slow during the seedling and vine stages, peaked at the flowering stage, and declined thereafter until maturity. During the flowering stage of pumpkins, there was a significant increase in water demand in, accounting for approximately one-third of the total ET. This could be attributed to the high temperature during this stage (in July), leading to strong transpiration and increased water demand for pumpkin fruit development. These findings align with previous studies (Zhou et al., 2020). Therefore, actual production should provide sufficient water during the flowering stage to ensure normal pumpkin growth and lay the foundation for high yield. The results indicated that increasing irrigation water and fertilizer led to higher ET in pumpkins throughout their growth period. This suggests that applying more organic fertilizer promoted pumpkin’s absorption and utilization of water, especially under lower irrigation volume. The application of W3 irrigation can significantly reduce ET by reducing fertilizer usage. However, the yield decreased under F3 treatment, and reducing fertilizer application not only reduced ineffective ET but also increased the yield. This maximizes the synergistic effect of coupling water and fertilizer, achieving the goal of transferring water with fertilizer and promoting fertilizer with water, the finding aligns with prior research (Fu et al., 2022; He et al., 2023a; Zhang et al., 2023c). In this experiment, There were a quadratic parabolic relationship between irrigation amount and water use efficiency (WUE), with the optimal irrigation amount enhancing WUE. However, when the irrigation level exceeded the critical value, WUE declined, consistent with previous studies (Yang et al., 2016; Jahromi et al., 2023). The experiment demonstrated that irrigation and fertilization had a positive coupling effect within the appropriate range, but excessive fertilization hindered efficient water use in pumpkin production, aligning with previous studies (Ye et al., 2022). The application amount is adjusted based on the irrigation volume in actual production, fully leveraging the coupling effect of water and fertilizer to improve water utilization and achieve high efficiency and high yield.

In this experiment, IUWE decreased with increasing irrigation volume, and initially increased but then decreased with the fertilization amount. This suggests that high irrigation and fertilization levels are not beneficial for water absorption and utilization in pumpkins. Under the F3W1 treatment, IWUE reached its maximum value, indicating that appropriate coupling scheme of water and fertilizer could improve water absorption and utilization in pumpkin plants (Piri and Albalasmeh, 2022; Zhang et al., 2023d).

The PFP exhibited a positive correlation with increasing irrigation amount, while showed a negative association with increasing fertilization amount (Zhang et al., 2017b; Da et al., 2023). A higher PFP did not necessarily result in the highest yield (Zhang et al., 2018). This experiment confirmed this finding, as it showed that F1W3 had the highest PFP, and a reduced yield deficit compared to the treatment F2W3. The scarcity of nutrients may cause pumpkin plants to prioritize their own growth by absorbing more, resulting in lower yields (Yue et al., 2023; He et al., 2023b).

In summary, the growth, yield, and water-fertilizer use efficiency of pumpkin were studied in this experiment under different water-fertilizer coupling control schemes. Principal component analysis and cluster analysis methods were used to comprehensively evaluate the different indices in 2022 and 2023. Both evaluation results were consistent, indicating that the F2W3 treatment ranked first in terms of comprehensive score. This treatment can serve as a valuable reference for high-yield and efficient pumpkin cultivation in the arid region of northwest China.

## 5 Conclusion

The response mechanism of pumpkin to the water-fertilizer coupling regulation was discussed by analyzing the variations in pumpkin growth and yield, ET, water and fertilizer use efficiency. The irrigation and fertilization levels, whether too low or too high, adversely affect pumpkin plant growth, yield, ET, water and fertilizer use efficiency. The higher fertilization level led to a decrease in pumpkin leaf area index and dry matter accumulation, inhibition of pumpkin yield, increased ET, and low water and fertilizer utilization efficiency. The use of organic fertilizer enhanced pumpkin growth, yield, WUE and IWUE compared to CK. The comprehensive evaluation of different water-fertilizer coupling treatments was conducted using principal component analysis and cluster analysis. The results indicated that F2W3 achieved the highest overall score in both 2022 and 2023. Therefore, the F2W3 treatment is recommended as the optimal water-fertilizer coupled scheme for pumpkin green production in the northwest arid region. It not only increases yield and efficiency but also promotes green environmental protection by saving fertilizers. The findings of this study are practically significant for enhancing crop yield and production efficiency in local and similar climate areas.

## Data availability statement

The original contributions presented in the study are included in the article/supplementary material. Further inquiries can be directed to the corresponding author.

## Author contributions

TZ: Writing – original draft, Writing – review & editing. JZ: Conceptualization, Data curation, Formal analysis, Funding acquisition, Methodology, Project administration, Resources, Supervision, Validation, Writing – review & editing. LLD: Conceptualization, Data curation, Formal analysis, Visualization, Writing – review & editing. LD: Funding acquisition, Investigation, Project administration, Resources, Supervision, Validation, Writing – review & editing. RZ: Conceptualization, Resources, Supervision, Writing – review & editing. XRL: Conceptualization, Methodology, Resources, Writing – review & editing. FR: Data curation, Methodology, Visualization, Writing – review & editing. MY: Data curation, Formal analysis, Software, Writing – review & editing. RY: Software, Visualization, Writing – review & editing. PT: Formal analysis, Visualization, Writing – review & editing. KG: Validation, Writing – review & editing. TY: Methodology, Writing – review & editing. QL: Resources, Software, Writing – review & editing. FL: Conceptualization, Supervision, Validation, Writing – review & editing. XL: Data curation, Supervision, Validation, Writing – review & editing.
